# Unveiling the rare: a case report of lumbar pseudoaneurysm secondary to tuberculous spondylitis

**DOI:** 10.1097/RC9.0000000000000191

**Published:** 2026-02-18

**Authors:** Arham Adnani, Lukas Widhiyanto

**Affiliations:** a Department of Orthopedics and Traumatology, Faculty of Medicine, Universitas Airlangga, Surabaya, Indonesia; b Department of Orthopedics and Traumatology, Dr. Soetomo General Academic Hospital, Surabaya, Indonesia

**Keywords:** case report, embolization, lumbar artery pseudoaneurysm, medicine, retroperitoneal abscess, tuberculous spondylitis

## Abstract

**Introduction and importance:**

Tuberculous spondylitis may compromise adjacent vascular structures, leading to the formation of tuberculous pseudoaneurysms – vascular lesions resulting from infection-induced destruction of the arterial wall. Despite their infrequency, these pseudoaneurysms carry a substantial risk of rupture and pose considerable diagnostic challenges.

**Case presentation:**

A 19-year-old female presented with a painful mass in the right abdominal region, persisting for 2 months. She had been diagnosed with tuberculous spondylitis involving the L1–L2 vertebrae. The initial management included posterior spinal debridement and stabilization from T12 to L4. Despite undergoing surgical intervention and anti-tuberculosis therapy for 8 months, the patient developed a firm, non-pulsatile mass in the right abdominal region. Imaging studies revealed a large retroperitoneal abscess and intraosseous collections. Abdominal CT angiography revealed a right lumbar artery pseudoaneurysm. The vascular lesion was successfully treated by embolization. This was followed by debridement and hematoma evacuation through a lumbotomy. No active bleeding from the pseudoaneurysm was observed during the intraoperative period.

**Clinical discussion:**

This case highlights the importance of maintaining a high index of suspicion for tuberculous pseudoaneurysms in patients with tuberculous spondylitis accompanied by adjacent abscesses. In such cases, clinicians should use advanced imaging modalities, including contrast-enhanced MRI or CT angiography, to improve diagnostic accuracy. Early detection of tuberculous pseudoaneurysms enables a staged therapeutic approach, beginning with prompt vascular repair to reduce the risk of rupture before spinal stabilization.

**Conclusion:**

Although tuberculous pseudoaneurysm is a rare complication of spinal tuberculosis, timely diagnosis and management are critical to preventing life-threatening sequelae.

## Introduction and importance

Spinal tuberculosis, or Pott’s disease, represents the most common form of osteoarticular tuberculous and accounts for approximately 1–2% of all TB cases[1]. While paravertebral cold abscesses and vertebral destruction are frequent in tuberculous spondylitis, the development of a tuberculous pseudoaneurysm – an abnormal dilation of the aortic wall due to infection-induced damage – is exceedingly rare, especially when associated with contiguous vertebral tuberculosis^[^2,3^]^.

HIGHLIGHTS

- Rare pseudoaneurysm complicates tuberculous spondylitis in a young female patient.
- CT angiography reveals lumbar artery pseudoaneurysm with retroperitoneal abscess.
- Embolization should be performed before definitive surgery.
- MRI or CTA is crucial when TB abscesses are near vascular structures.
- Early detection and staged surgery with TB therapy improve outcomes.

Tuberculous pseudoaneurysms, often saccular and pseudo in nature, typically result from erosion of the aortic wall by adjacent infectious foci, such as paravertebral abscesses or lymphadenitis[4].

This case highlights the rare occurrence of a lumbar artery pseudoaneurysm in a patient with tuberculous spondylitis and a retroperitoneal abscess, emphasizing the need for clinical suspicion and comprehensive imaging in patients with tuberculous spondylitis and vascular structures. This case report has been reported in line with the SCARE 2025 criteria[5].

## Case presentation

A 19-year-old female student presented with a 2-month history of a painful lump accompanied by a burning sensation localized to the right side of her abdomen. Approximately 9 months prior to presentation, she began experiencing persistent lower back pain associated with paresthesia in the lower extremities.

**Figure 1.**
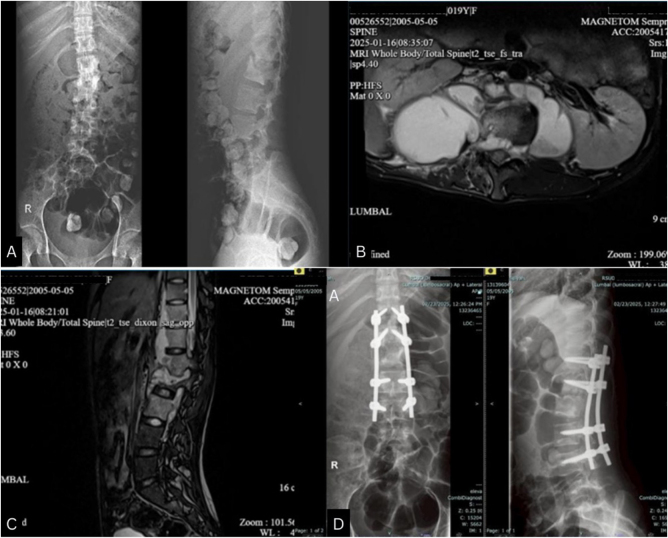
(A–C) Preoperative Radiology that shows tuberculous spondylitis involving the L1–L2 vertebrae and paravertebral abscess; (D) Postoperative Radiology that shows spinal instrumentation after debridement and posterior stabilization.

Six months prior, she was diagnosed with tuberculous spondylitis and subsequently initiated on standard anti-tuberculosis therapy. Three months prior, the patient had undergone posterior debridement and stabilization from T12 to L4 due to tuberculous spondylitis involving the L1–L2 vertebrae. Intraoperatively, approximately 2 liters of purulent material was encountered. Postoperatively, neurological evaluation revealed no deficits, indicating preserved motor function and intact sensory modalities (Fig. 1).

**Figure 2.**
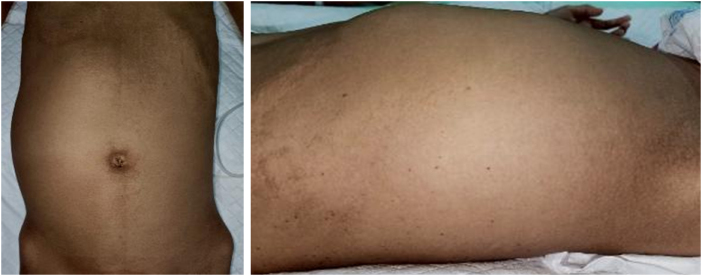
Clinical picture that shows distended abdomen and palpated mass in the right lumbar region.

On physical examination, the abdomen appeared mildly distended, and a firm, fixed, and mass measuring approximately 20 × 10 cm was palpated in the right lumbar region. No indications of urinary or bowel dysfunction were observed (Fig. 2). The laboratory examination showed erythrocyte sedimentation rate (ESR) 70 mm/hour, C-reactive the following: white blood cell count (WBC) 5.6 × 10^9^ cells/l, protein (CRP) 0.87 mg/l, hemoglobin 6.5 g/l, albumin 2.70 g/l, and HIV-ab negative.

**Figure 3.**
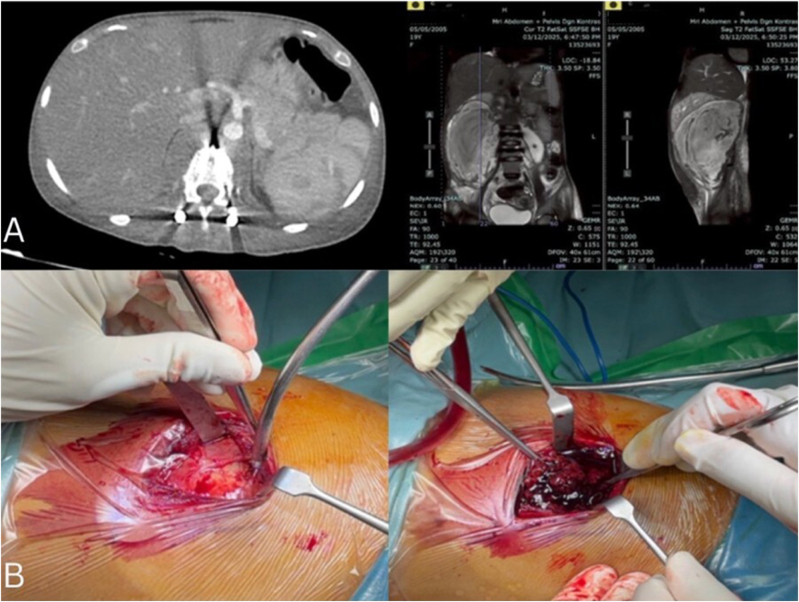
(A) MRI and CT, demonstrated destruction of the L1–L2 vertebral bodies and a large retroperitoneal abscess; (B) First intraoperative picture surgery that shows clot formation in the right abdomen.

Imaging studies, including MRI and CT, demonstrated destruction of the L1–L2 vertebral bodies and a large retroperitoneal abscess measuring up to 19.8 cm in diameter. The abscess extended into both psoas muscles, the paravertebral space, and the subcutaneous tissues from T12 to L4 (Fig. 3). The patient was initially diagnosed with tuberculous spondylitis L1-2 with paravertebral and right psoas abscesses. Debridement via the Lumbotomy Approach was performed to evacuate the abscess. However, the surgery for debridement was postponed due to intraoperative findings of clot formation (Fig. 3b). We suggest that another examination be performed.

**Figure 4.**
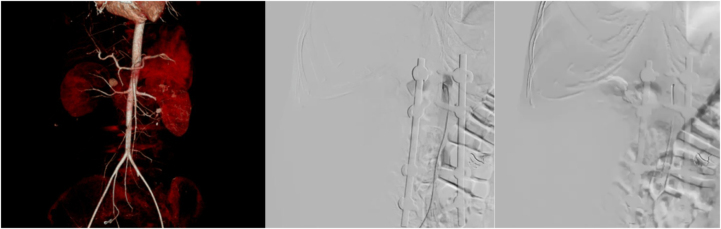
CT Angiography demonstrated a right lumbar artery pseudoaneurysm and embolization of the pseudoaneurysm was performed.

Abdominal CT angiography revealed a vascular lesion measuring approximately 1.3 × 1.5 × 1.1 cm, located posterior to the right renal artery at the L1 level, with a surrounding hematoma, highly suggestive of a lumbar artery pseudoaneurysm (Fig. 4).

**Figure 5.**
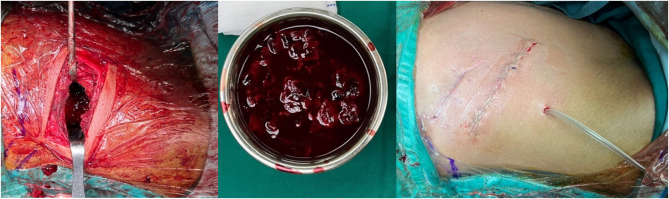
Intraoperative picture of the second surgery that shows clot formation evacuation.

Subsequently, embolization of the right lumbar artery pseudoaneurysm was successfully performed after intraoperative confirmation of active extravasation at L1. The patient underwent debridement via a lumbotomy approach for the second time, along with evacuation of the clot. No active bleeding was observed in the pseudoaneurysm. Approximately, 1.5 liters of the clot was successfully evacuated (Fig. 5).

## Discussion

Tuberculous pseudoaneurysms may form through several mechanisms, including bacterial spread via lymphatic vessels surrounding the arteries, direct inoculation of *Mycobacterium tuberculosis* following vascular injury, invasion of the vessel wall through feeding arteries, or direct extension from adjacent infected lymph nodes, abscesses, or tuberculous bone lesions near the artery[4].

Most patients exhibited multiple clinical symptoms, with the following common presentations: persistent pain, fever, signs of blood volume depletion or hemorrhagic shock, and noticeable mass. Pain and the presence of a mass are the most frequently observed symptoms of this disease. In this case, the patient’s symptoms were related to the pseudoaneurysm, specifically back pain, progressive anemia, and a large mass identified in the right abdomen. However, apart from a few large aneurysms presenting as visible or palpable pulsatile masses, most pseudoaneurysms lack distinct clinical features and are often misdiagnosed[4].

Spinal tuberculosis is diagnosed through a combination of clinical symptoms and confirmation of Mycobacterium tuberculosis. Although X-rays can detect spinal tuberculous with up to 99% accuracy, they are limited in evaluating the full extent of vertebral destruction, spinal cord compression, and the presence of abscesses or pseudoaneurysms. Therefore, CT or MRI scans are considered the most effective diagnostic tools for identifying tuberculous pseudoaneurysms[6].

In the present case, preoperative MRI showed a paravertebral abscess, but without CT angiography, the pseudoaneurysm was not identified, leading to its rupture during surgery. We suggest that if a paravertebral abscess is found adjacent to the aortic vessels on MRI, further evaluation with CT angiography should be conducted to rule out or confirm a pseudoaneurysm. In this case, based on the patient’s initial clinical symptoms and findings from CT and MRI, tuberculous involvement was identified around the pseudoaneurysm, along with an associated, large abscess.

Currently, the recommended approach for managing this condition involves surgical intervention combined with concurrent anti-tuberculosis therapy. This is particularly important given the lack of evidence supporting the effectiveness of anti-TB medication or surgery alone in achieving a cure for this condition[7]. Currently, aneurysms are treated through either open surgical procedures – such as direct suture closure, excision of the vascular lesion with synthetic graft replacement, – or through minimally invasive techniques, including embolization, endovascular stent-graft placement, and endovascular aneurysm repair (EVAR)[6].

The management of tuberculous pseudoaneurysm requires a combination of prompt surgical intervention and anti-tuberculosis therapy^[^2,8^]^. Surgical options include open repair with in situ graft placement or extra-anatomic bypass, and more recently, endovascular aneurysm repair (EVAR) has emerged as a promising alternative^[^4,9^]^.

In this case, a pseudoaneurysm of the right lumbar artery was successfully managed with endovascular embolization in conjunction with debridement and spinal stabilization. This approach aligns with current recommendations favoring staged interventions, where aneurysm repair precedes spinal surgery to minimize the risk of rupture^[^4,7^]^.

A tuberculous pseudoaneurysm associated with spinal tuberculosis is a rare condition that presents significant diagnostic and therapeutic challenges. Achieving optimal outcomes requires a high index of suspicion, timely application of advanced imaging, and a coordinated multidisciplinary approach that integrates surgical and medical management (3,4). In this case, a large clot was discovered intraoperatively during the initial abscess debridement, leading to postponement of the surgery. Subsequent CT angiography confirmed a right lumbar artery pseudoaneurysm, which was successfully managed by endovascular embolization under anesthesia. Although initially anxious when her abdominal lump enlarged and surgery was delayed, the patient felt reassured after the diagnosis and treatment plan were explained, and she recovered without further complications.

This case report highlights a very rare presentation of lumbar artery pseudoaneurysm secondary to spinal tuberculosis, supported by detailed imaging, surgical findings, and staged management. The multidisciplinary approach applied in this case underlines the importance of integrating vascular intervention with infection control to achieve favorable outcomes. However, the follow-up period was relatively short (6 months), which limits the ability to draw long-term conclusions regarding recurrence or durability of treatment. Furthermore, as a single case, the findings cannot be generalized to all patients with tuberculous spondylitis.

## Patient perspective

The patient initially felt anxious when the abdominal lump enlarged and surgery was delayed, but after receiving an explanation of the diagnosis and treatment plan, she felt reassured and expressed satisfaction with her recovery.

## Conclusion

A tuberculous pseudoaneurysm of the lumbar artery associated with spinal tuberculosis is rare but has severe complications. When a paravertebral abscess is detected near the thoracic aorta on preoperative CT scans, further evaluation with MRI or CT angiography (CTA) is recommended to rule out the presence of a pseudoaneurysm. Once diagnosed, surgical intervention must be undertaken, in combination with anti-tuberculosis therapy, regardless of the pseudoaneurysm size, to prevent the risk of sudden and potentially serious complications.

## Data Availability

Not applicable.
